# Development of the spatial contrast sensitivity function (CSF) during childhood: Analysis of previous findings and new psychophysical data

**DOI:** 10.1167/jov.20.13.4

**Published:** 2020-12-04

**Authors:** Tessa M. Dekker, Mahtab Farahbakhsh, Janette Atkinson, Oliver J. Braddick, Pete R. Jones

**Affiliations:** Child Vision Lab, Institute of Ophthalmology, University College London (UCL), London, UK; NIHR Moorfields Biomedical Research Centre, London, UK; Division of Optometry and Visual Science, City, University of London, London, UK; Faculty of Brain Sciences, University College London (UCL), London, UK; Department of Experimental Psychology, University of Oxford, Oxford, UK; Division of Psychology and Language Sciences, University College London (UCL), London, UK

**Keywords:** children, development, contrast sensitivity, contrast sensitivity function, psychophysics, QUEST+, Weighted Staircase, Gabor, four alternative forced choice

## Abstract

Although the contrast sensitivity function (CSF) changes markedly during infancy, there is no consensus regarding whether, how, and why it continues to develop in later childhood. Here, we analyzed previously published data (*N* = 1928 CSFs), and present new psychophysical findings from 98 children (4.7–14.8 years) and 50 adults (18.1–29.7 years), in order to answer the following questions: (1) Does the CSF change during childhood? (2) How large is the developmental effect size? (3) Are any changes uniform across the CSF, or frequency-specific? and (4) Can some or all of the changes be explained by “non-visual” (i.e. procedural/cognitive) factors, such as boredom or inattentiveness? The new data were collected using a four-alternative forced-choice (4AFC) Gabor-detection task, with two different psychophysical procedures (Weighted Staircase; QUEST+), and suprathreshold (false-negative) catch trials to quantify lapse rates. It is shown that from ages 4 to 18 years, the CSF improves (at an exponentially decaying rate) by approximately 0.3 log_10_ units (a doubling of contrast sensitivity [CS]), with 90% of this change complete by 12 years of age. The size of the effect was small relative to individual variability, with age alone explaining less than one sixth of variability (16%), and most children performing as well as some adults (i.e. falling within the 90% population limits for adults). Development was frequency-specific, with changes occurring primarily around or below the CSF peak (≤ 4 cpd). At least half — and potentially all — of the changes observed could be explained by non-visual factors (e.g. lapses in concentration), although possible biological mechanisms are discussed.

## Introduction

Knowing how the spatial contrast sensitivity function (CSF) changes from birth to adulthood is important for understanding the development of normal visual capacities. It is also important for establishing useful benchmarks against which to compare clinical populations, given that the CSF is affected in many childhood visual disorders, including amblyopia (Sjöstrand, 1981; Howell, Mitchell, & Keith, 1983; Koskela & Hyvarinen, 1986; Wang, Zhao, Ding, & Wang, 2017), optic neuritis (Zimmern, Campbell, & Wilkinson, 1979), congenital hypothyroidism (Mirabella et al., 2005), retinitis pigmentosa (Hyvärinen, 1983), cataract (Vasavada et al., 2014), corneal edema (Hess & Garner, 1977), and cerebral lesions (Bodis-Wollner & Diamond, 1976) (for an overview, see Milling, O'Connor, & Newsham, 2014).

During infancy, the CSF is known to undergo substantial change (Atkinson, Braddick, & Braddick, 1974; Banks & Salapatek, 1976; Harris, Atkinson, & Braddick, 1976; Atkinson, Braddick, & Moar, 1977; Pirchio, Spinelli, Fiorentini, & Maffei, 1978; Banks & Salapatek, 1981; Atkinson & French, 1983; Movshon & Kiorpes, 1988; Norcia, Tyler, & Hamer, 1990; Adams & Courage, 1993; Peterzell, Werner, & Kaplan, 1995; Allen, Tyler, & Norcia, 1996; Gwiazda, Bauer, Thorn, & Held, 1997; Hainline & Abramov, 1997). The overall shape of the function (an “inverse U”) remains roughly constant from 2 to 3 months onward (Atkinson, Braddick, & Moar, 1977), but peak sensitivity increases approximately 10-fold during the first year of life (Norcia, Tyler, & Hamer, 1990; Peterzell, Werner, & Kaplan, 1995) (a large upward shift of the curve), and peak frequency increases also (a modest rightward shift of the curve) (Pirchio, Spinelli, Fiorentini, & Maffei, 1978; Movshon & Kiorpes, 1988; Peterzell, Werner, & Kaplan, 1995; Hainline & Abramov, 1997). These changes are thought to reflect progressive maturation in retinal organization and further visual pathways (Wilson, 1988; Banks & Crowell, 1993; Kiorpes, Tang, Hawken, & Movshon, 2003).

There is no consensus, however, regarding whether or not the CSF continues to develop during later childhood (Table 1). Some studies suggest that the CSF remains immature at 4 to 5 years (Atkinson, French, & Braddick, 1981; Ellemberg, Lewis, Hong Liu, & Maurer, 1999), but is largely adult-like by around 6 to 8 years (Derefeldt, Lennerstrand, & Lundh, 1979; Bradley & Freeman, 1982; Abramov et al., 1984; Adams & Courage, 2002). Other studies report a more protracted developmental trajectory, with the CSF continuing to shift upward until late childhood (Scharre, Cotter, Block, & Kelly, 1990; Benedek, Benedek, Kéri, & Janáky, 2003) or even into adolescence (Arundale, 1978; Almoqbel, Irving, & Leat, 2017). Thus, it is not uncommon to read on the one hand that “contrast sensitivity and acuity, measured psychophysically, are mature by 5 to 6 years in humans” (Kiorpes, Tang, Hawken, & Movshon, 2003), but also that “contrast sensitivity is still not adult-like by 8 years” and may “mature fully between the ages of 8 to 19 years” (Leat, Yadav, & Irving, 2009). Studies using optotype charts have likewise been often inconclusive, but indicate that contrast sensitivity (CS) may continue to develop after 6 to 8 years (Leat & Wegmann, 2004; Hargadon et al., 2010) and potentially into adulthood (Mäntyjärvi & Laitinen, 2001).

**Table 1.** Summary of studies that measured CSFs in children using static gratings (Mayer, 1977; Arundale, 1978; Derefeldt, Lennerstrand, & Lundh, 1979; Beazley, Illingworth, Jahn, & Greer, 1980; Atkinson, French, & Braddick, 1981; Bradley & Freeman, 1982; Abramov et al., 1984; Scharre, Cotter, Block, & Kelly, 1990; Gwiazda, Bauer, Thorn, & Held, 1997; Ellemberg, Lewis, Hong Liu, & Maurer, 1999; Adams & Courage, 2002; Benedek, Benedek, Kéri, & Janáky, 2003; Almoqbel, Irving, & Leat, 2017; Cornick, Hallett, Higgins, & Drover, 2017). Shaded blocks (column 4) indicate the ages of children tested, and whether performance was adult-like (green), or differed significantly from adult controls (red). In the case of Abramov et al. (1984) observed performance was significantly poorer than adults, but the authors dismissed the difference as a procedural artifact.

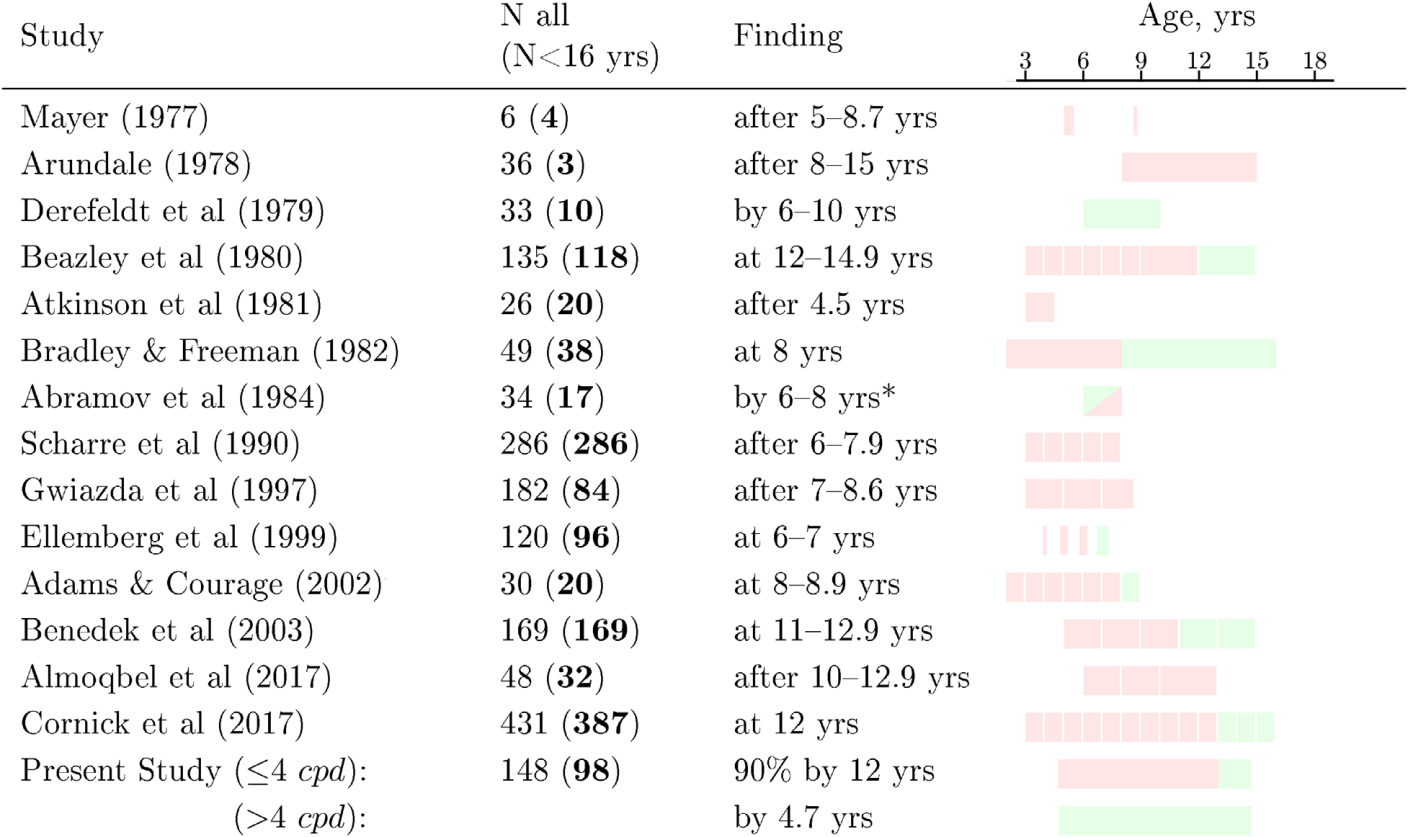

There is also disagreement about whether any changes during childhood affect the whole CSF, or whether certain spatial frequencies see improvements in sensitivity with age. Thus, some authors have reported a relatively uniform increase of CS at all spatial frequencies (Scharre, Cotter, Block, & Kelly, 1990; Ellemberg, Lewis, Hong Liu, & Maurer, 1999; Cornick, Hallett, Higgins, & Drover, 2017), whereas others report changes at low-frequencies only (Bradley & Freeman, 1982; Adams & Courage, 2002), and others still have reported changes at all frequencies but with greatest change at low frequencies (Gwiazda, Bauer, Thorn, & Held, 1997; Beazley, Illingworth, Jahn, & Greer, 1980; Benedek, Benedek, Kéri, & Janáky, 2003). These differing conclusions may reflect a range of factors, including random measurement error, differences in the stimuli or spatial frequencies tested, differences in psychophysical methods, and/or differences in statistical power.

Finally, it is also unclear to what extent any developmental effects represent genuine changes in perception, versus “non-visual” factors, such as boredom or lapses in concentration. The latter are always key potential confounds when comparing any psychophysical measurements in children (Westall et al., 1992; Wightman & Allen, 1992; Witton, Talcott, & Henning, 2017; Jones, 2018; Manning, Jones, Dekker, & Pellicano, 2018), and have been hypothesized previously to explain some or all developmental CSF changes, post-infancy (Bradley & Freeman, 1982; Abramov et al., 1984). Again, however, the literature is divided, with some authors asserting that “these differences [in CSF] are due to anatomical or physiological visual development, rather than behaviour” (Almoqbel, Irving, & Leat, 2017), whereas others state that “nonneural factors, probably related to the capacity to attend the task, may limit the visual performance of very young children” (Bradley & Freeman, 1982).

To understand the overall size and shape of the developmental effect, in the present work we analyzed data extracted from all known previously published studies (studies listed in Table 1). We also analyzed new data collected from 98 children (4.7–14.8 years) and 50 young adults (18.1–29.7 years), all of whom were asked to complete two psychophysical assessments using a “gold standard” four-alternative forced-choice (4AFC) Gabor detection task. To ensure robustness, this dataset included convergent measurements made using two different psychophysical techniques: a conventional staircase procedure (with independent staircases performed at each spatial frequency), and a maximum likelihood procedure, adjusting the parameters of an overall CSF (QUEST+) (Watson, 2017). Unusually, we also tested spatial frequencies up to 30 cycles-per-degree (cpd) in order to examine how the CSF develops at higher frequencies, and we consider individual data and data from suprathreshold (false-negative) catch trials in order to examine whether any developmental effects are due to a small minority of “non-compliant” children (e.g. children whose compliance varied due to boredom or lapses in concentration).

The goal of the present study was to answer the following four questions: (1) Does the CSF change during childhood? (2) How large is the developmental effect size? (3) Are any changes uniform across the CSF, or frequency-specific? (4) Can some or all of the changes be explained by “non-visual” (i.e. procedural/cognitive) factors, such as boredom or inattentiveness?

## Methods

### Analysis of previously published data

Data were extracted from the relevant tables and figures of the studies listed in Table 1. To ensure accuracy, values were entered independently by two people (authors M.F. and P.J.). The dataset of extracted values is available as Supplementary Data.

### New empirical data

#### Overview

CSFs were measured for 98 children (4.7–14.8 years) and 50 adults (18.1–29.7 years).

#### Participants

Experiment 1 examined 71 children (aged 4.7–14.7 years; 75% within ages 6–11.5 years) and 43 young adults (ages 18.1–29.6 years). The full distribution of ages is shown graphically in the Supplementary Methods. Elements of these data have been reported previously when considering the performance of different psychophysical algorithms in children (Farahbakhsh, Dekker, & Jones, 2019). However, the developmental effects reported in the present paper have not been previously analyzed or reported. Experiment 2 consisted of new, previously unreported data from an additional 27 children (aged 8.3–14.8 years) and 7 young adults (aged 18.9–29.7 years), none of whom participated in experiment 1.

All participants were required to have normal or corrected-to-normal vision, as defined by no reported history of eye disease, and a binocular letter acuity score of 0.16 logMAR (6/9) or better as assessed using an ETDRS chart (early treatment diabetic retinopathy study) at 4 meters (Precision Vision Ltd., La Salle, IL, USA). Three additional children were recruited, but their data are not reported as they did not pass the screening criteria for normal vision.

Adults were recruited through the UCL Psychology Subject Pool (“SONA”), and received £7/hour compensation. Children were recruited through the UCL Child Vision Lab volunteer database, and received certificates, small toys, and transportation costs. Informed written consent was obtained from all adults and parents, and children gave verbal assent. The research was carried out in accordance with the tenets of the Declaration of Helsinki, and was approved by the UCL Ethics Committee (#1153/001).

#### Stimuli

##### Experiment 1

The target stimulus was a horizontal Gabor patch of variable contrast and spatial frequency. The standard deviation of the Gaussian hull was 0.14 degrees visual angle, meaning that 99% of the stimulus energy fell within a diameter of 0.72 degrees. The total diameter (“mathematical support”) of the Gabor was 1 degree (seven times its standard deviation). Stimulus duration was 500 ms, including 83 ms raised-cosine on/off ramps. The mean luminance of the Gabor was 136 cd/m^2^, and it was presented against an equiluminant gray background. On each trial, a single Gabor was randomly presented at one of four cardinal locations (north, south, east, or west), with the center of the Gabor always located 1.5 degrees eccentric from a central fixation cross. Immediately following stimulus presentation, white noise masks were displayed for 100 ms at all 4 potential target locations. Participants were then given unlimited time to indicate the location of the Gabor by pressing one of four arrows on a keypad. Participants generally pressed the response buttons themselves, although occasionally the experimenter would press the button for a period under instruction from the participant (i.e. if the participant appeared to be becoming inattentive). After a response was entered, veridical auditory and visual feedback was presented for 200 ms, in the form of a happy/sad cartoon zebra and a corresponding sound. The next trial then commenced automatically after an inter-trial interval of 100 ms. The contrast and spatial frequency of the stimulus on each trial was determined by the psychophysical algorithm, details of which are described below. Participants were encouraged to take breaks whenever they felt the need to do so.

##### Experiment 2

The target stimulus in experiment 2 was identical to that in experiment 1, with the following exceptions. The standard deviation of the gaussian hull was increased from 0.14 degrees to 0.7 degrees (⌀_99%_ = 3.6 degrees, ⌀_Total_ = 7.0 degrees), in order to accommodate lower spatial frequencies. To maintain a similar eccentricity, the distance from the central fixation cross to the middle of each Gabor was also increased from 1.5 degrees to 3.0 degrees. This resulted in the most central portion of the stimulus being somewhat closer to the midline than in experiment 1. The mean luminance of the Gabor (and thus the equiluminant background as well) was also reduced to 50 cd/m^2^, as these data were intended to serve as a benchmark for future clinical studies involving patients with photophobia.

#### Apparatus

Stimuli were presented on a 27 inch 10-bit LCD (IPS) monitor (EIZO ColorEdge CG2730; 2560 × 1440 pixels; EIZO Co., Ltd., Birmingham, UK), connected via DisplayPort to a 10-bit graphics card (Nvidia GeForce GTX 650Ti; Nvidia Corp., Santa Clara, CA, USA). The screen was viewed binocularly at a distance of approximately 160 cm. Although viewing distance was not precisely controlled, children were closely monitored, and no substantive head movements were observed. Head movements greater than approximately ± 10 cm would have caused the eye-tracker to stop tracking, and this never occurred in practice.

Throughout the experiment, participants were reminded regularly to fixate the central fixation target, which consisted of a black circle, 0.19 degrees in diameter, always visible in the middle of the screen. To ensure compliance, gaze location was monitored continuously using a remote eye tracker (Tobii X120; Tobii Technology AB, Danderyd, Sweden). If at any point the participant's gaze deviated by more than 2 degrees from the central fixation spot, the experiment automatically paused and the fixation point turned gray.

Hardware were controlled using custom MATLAB code (R2016b, MathWorks, Natick, MA, USA), via the Psychophysics Toolbox 3 (Brainard, 1997; Kleiner et al., 2007) and Tobii SDK 3.0 (Tobii Technology AB, Danderyd, Sweden). The monitor was calibrated using a ColorCal Mk.2 colorimeter (Cambridge Research Systems, Cambridge, UK) and the calibration was validated using a Minolta CS-100 photometer (Minolta Camera Co., Osaka, Japan), and also by the monitor's own integrated photometer (EIZO Co., Ltd., Birmingham, UK).

Testing took place in a quiet room under mesopic illumination (12.6 lx; Amprobe LM-120 Light Meter; Danaher Corporation, Washington DC, USA). Participants were seated throughout the test. Accompanying adults were encouraged to leave the room during testing to avoid distracting the participant. When present (in approximately 10% of cases), adults sat outside the child's eyeline, and were asked to remain silent during testing. To avoid any potential distractions, the area around the participant and screen was separated from the main room by a thin black cotton curtain.

#### Procedure

Participants performed a 4AFC Gabor detection task, presented as a game in which participants were asked to “find where the zebra is hiding.”

Experiment 1 measured CSFs between 2 and 30 cpd (2, 4, 8, 10, 16, 20, 25, and 30), using 2 different psychophysical methods: (i) a conventional staircase procedure in which CS was measured independently at 8 discrete spatial frequencies, and (ii) a novel, QUEST+ (Watson, 2017) maximum likelihood (ML) procedure similar to the “quick CSF” (qCSF) (Hou et al., 2010; Lesmes, Lu, Baek, & Albright, 2010; Rosén et al., 2014) in which contrast and spatial frequency were adapted simultaneously in order to directly fit a single overall CSF. Further details of these psychophysical procedures are presented in the Supplementary Methods. All participants attempted to complete two CSF assessments, either: 2 × staircase (*N* = 21 children, 15 adults), 2 × ML (*N* = 16 children, 15 adults), or one of each (*N* = 34 children, 13 adults). However, as detailed previously (Farahbakhsh, Dekker, & Jones, 2019), some participants ultimately contributed data for only one assessment, either because they were too young to complete two assessments (*N* = 12 children), or due to technical errors in the initial implementation of the ML procedure leading to invalid/unusable data (*N* = 6 children). In practice, the data from the two psychophysical methods were highly correlated (see Supplementary Results), so all CSFs were averaged within-subjects, to yield one CSF estimate per observer (114 CSFs total).

Experiment 2 measured CSFs between 0.5 and 30 cpd (0.5, 1, 2, 3, 4, 8, 15, and 30), using a staircase procedure only. The ML procedure was not used in experiment 2, as we were not confident that we could specify, a priori, the correct parametric shape of the CSF at low frequencies, which is known to differ markedly between observers (Rohaly & Owsley, 1993). All of the 27 children and 7 adults in experiment 2 completed one CSF assessment only (34 CSFs total), although, unlike experiment 1, extensive practice was provided beforehand (see below).

Furthermore, in both experiments, and regardless of the psychophysical algorithm, approximately 30 additional catch trials were interleaved throughout the test. These were uniformly randomly distributed once every 6 test trials (ML method), or once every 13 test trials (Staircase method). The stimulus on these catch trials consisted of a highly suprathreshold Gabor (spatial frequency: 3–16 cpd; Michelson contrast = 0.8–1.0), which was expected to be clearly visible to all participants. These trials were intended to quantify lapse rates and motivate participants only, and were not used when computing CSFs. Mathematically, lapse rates were computed as: 100*(m*FN)/(*m*-1), where *FN* was the proportion of false-negative responses, and *m* was the number of response alternatives (*m* = 4).

Trials were divided into blocks (“levels” of the game). In the Staircase condition, there were eight blocks: each corresponding to a single adaptive-track/spatial-frequency. Staircase blocks were of variable length, but on average consisted of 47 trials including catch trials (approximately 376 trials total). In the ML condition, there were 6 blocks: each consisting of exactly 35 trials, including catch trials (210 trials total), and the same instance of the algorithm ran continuously across all blocks. Participants were encouraged to take short breaks between blocks as required (generally less than a minute).

Prior to testing, participants completed 2 practice blocks of 9 and 15 trials. During the first practice block, the target Gabor continued to remain visible until the participant responded, and the experimenter pressed the response key for the first three trials. This block was to familiarize the participants with the game. In the second practice block, the trials were identical to the main experiment, however, a fixed sequence of stimulus levels was used, designed to demonstrate a representative range of possible frequency/contrast levels, including both sub- and suprathreshold magnitudes. The criterion for completing the 2 practice blocks successfully was ≥ 90% correct responses on those trials expected to be suprathreshold (maximum contrast). Most participants achieved this on their first attempt (experiment 1: 97%; and experiment 2: 100%). In experiment 1, three individuals failed to reach this criterion on their first attempt, and so repeated both practice blocks, at which point the criterion was met by all three of these participants. In experiment 2, participants also completed an extensive additional practice session (mean = 331 trials), to minimize any possible learning effects.

The total duration of the experiment was approximately 60 minutes (experiment 1) or 30 minutes (experiment 2), including breaks. Extensive efforts were made to ensure that the children understood the task and remained focused and engaged throughout testing. This included the use of: trial-by-trial feedback, frequent suprathreshold “motivational” trials (see above), regular breaks, an engaging “zebra safari” story with colorful graphics (these additional graphics were presented between test blocks), carefully structured practice trials, gaze monitoring via a remote eye-tracker, nonspecific encouragement from the experimenter (author M.F.), and the regular awarding of “points” that could be exchanged for small toys at the end of the game.

## Results

### Does the CSF change during childhood?

**Figure 1.**
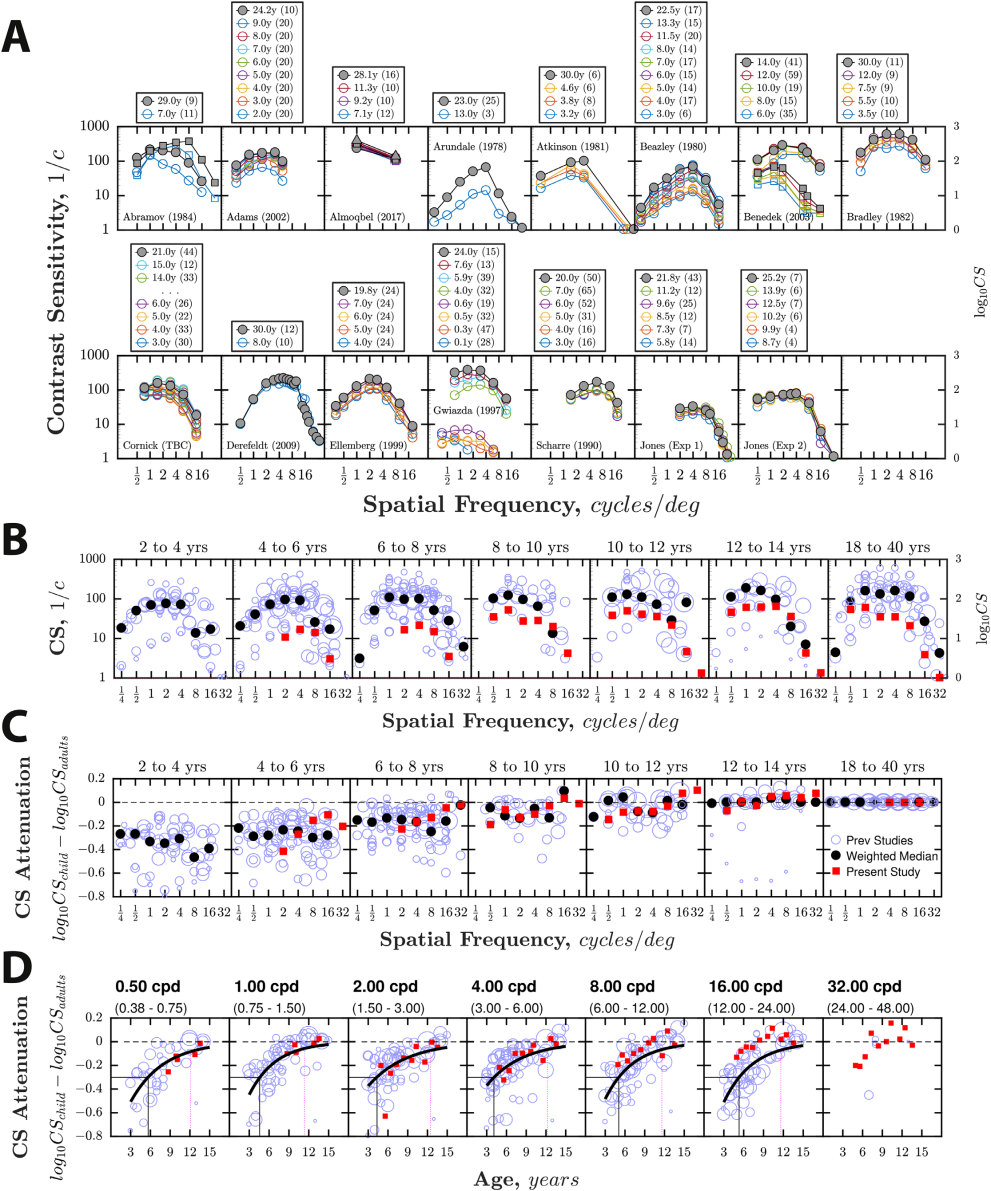
CSF data from previous and present experiments, plotted four ways (same data in each panel). (**A**) CSFs by study. The adult age group is highlighted in gray. For Almoqbel (2017) and Benedek (2003), the different markers indicate different experimental conditions (see Supplemental Data for details). (**B**) CSFs aggregated across all studies. Blue circles indicate group-mean data (size proportional to sample size). Black filled circles show the weighted median^1^ of all previous studies, weighted relative to sample size. Red filled squares are the group-mean values from the present study (combining data across both experiments). (**C**) Same as **B** but showing the log difference between adults and children (“CS Attenuation”), computed independently within each study. The horizontal dashed line indicates zero (adult-like performance). Note that by definition the values in the 18- to 40-year-old group will be zero. These data were therefore not included in any statistical analyses. (**D**) Same as **C** but plotted as a function of age, using 1-year wide age bins. The black line indicates the least-square exponential curve [y = a⋅*e*^(b⋅x)^], fitted to children's data from previous studies, with each data point weighted proportional to sample size. The vertical black solid line indicates the age at which performance was 0.3 log_10_ units below adults, which occurred between 4.1 and 5.7 years (mean = 4.8 years). The vertical magenta dotted line indicates the point on the curve between 4 and 18 years by when CS had improved by 90% (11.4–12.5 years; mean = 11.9).

CSF data from all previous studies are summarized in Figure 1. Although substantial interstudy variability is evident, group-mean CS approximately doubled (i.e. improved by approximately 0.3 log_10_ units) between 4 and 18 years (0.28–0.39 log_10_ units, depending on frequency; mean = 0.34). Within this period, the rate of change was well described by an exponential decay curve (increasing form), with 90% of the change after 4 years complete by 12 years (Figure 1D; mean = 11.9 years; range = 11.4–12.5 years), and 75% by 9 years (mean = 9.3 years; range = 8.8–9.9 years).

The combined data from the two present experiments are also shown in Figure 1. Absolute CS values were at the lower limit of those previously published (see Figure 1A,B). This is likely due to the fact that the stimuli were paracentral and fixed duration (i.e. values were consistent with previous adult data for paracentral grating stimuli, as discussed previously elsewhere; Farahbakhsh, Dekker, & Jones, 2019). However, the relative difference between children and adults remained similar at low spatial frequencies. Thus, in terms of the overall size and shape of the developmental effect, the present data appeared to be in good agreement with previous studies at frequencies ≤ 4 cpd (Figure 1D). However, the developmental effect did not appear uniform across spatial frequencies. Thus, although Figure 1D shows a trend of CS increasing with age across the range of spatial frequencies tested, above 4 cpd the developmental changes appeared smaller, and in many cases were statistically nonsignificant (see below for further details and formal analysis).

As shown in Table 2, we were able to demonstrate that the associations between CS and age in the present dataset persisted if we: (1) considered only data from the Staircase or ML method; (2) considered only the first CSF assessment, to control for possible fatigue effects; (3) considered only the second CSF assessment, to control for possible learning effects; (4) excluded the youngest children (those less than 6.0 years old; *N* = 8); or (5) excluded statistical outliers (those more than three times the Median Absolute Distance from the group-median; *N* = 0–17, depending on spatial frequency).

**Table 2.** Correlation between contrast sensitivity and age, broken down by spatial frequency (columns) and method of analysis (rows). Instances where a significant (*p* < 0.05) correlation was observed are highlighted in bold. Note that the *p* values in the first and last row are also given in Figure 2.

	Spatial frequency, *cpd*																	
	2		4		8		10		16		20		25		30		AUCSF	
Analysis method	*p* value	r	*p* value	r	*p* value	r	*p* value	r	*p* value	r	*p* value	r	*p* value	r	*p* value	r	*p* value	r
Standard (mean of both tests, *Spearman corr*)	<0**.001**	0.54	<0**.001**	0.41	**0** **.014**	0.23	0.073	0.17	0.318	0.09	0.360	0.09	0.142	0.14	0.151	0.14	**0** **.003**	0.27
Staircase tests only	<0**.001**	0.47	**0** **.006**	0.30	0.259	0.13	0.523	0.07	0.921	0.01	0.724	0.04	0.713	0.04	0.085	0.19	0.126	0.17
ML tests only	<0**.001**	0.49	<0**.001**	0.42	0.060	0.21	0.243	0.13	0.659	0.05	0.561	0.07	0.425	0.09	0.348	0.11	0.052	0.22
First test only	<0**.001**	0.45	<0**.001**	0.33	0.091	0.16	0.324	0.09	0.523	0.06	0.422	0.08	0.556	0.06	0.421	0.08	**0** **.027**	0.21
Second test only	<0**.001**	0.52	<0**.001**	0.37	0.097	0.17	0.141	0.15	0.798	0.03	0.823	0.02	0.244	0.12	0.015	0.25	**0** **.017**	0.24
Young children excluded (< 6:0 years)	<0**.001**	0.49	<0**.001**	0.34	0.082	0.17	0.261	0.11	0.708	0.04	0.537	0.06	0.678	0.04	0.585	0.05	**0** **.042**	0.20
Outliers excluded (>3 x MAD)	<0.**001**	0.51	<0**.001**	0.37	**0** **.030**	0.22	0.181	0.13	0.274	0.11	0.360	0.09	0.213	0.12	0.816	0.02	**0** **.005**	0.27
High lapse rate excluded (>10%)	<0**.001**	0.48	<0**.001**	0.34	0.152	0.15	0.454	0.08	0.857	0.02	0.866	0.02	0.791	0.03	0.387	0.09	0.097	0.17
Adjusting for lapse rate (partial *Spearman corr*)	<0**.001**	0.40	**0** **.006**	0.26	0.442	0.07	0.978	0.00	0.558	0.06	0.302	0.10	0.896	0.01	0.332	0.09	0.326	0.09

For interested readers, data for individual observers are also given in Supplementary Results, along with evidence of good test-retest agreement between the two psychophysical methods used in experiment 1.

### How large is the developmental effect size?

**Figure 2.**
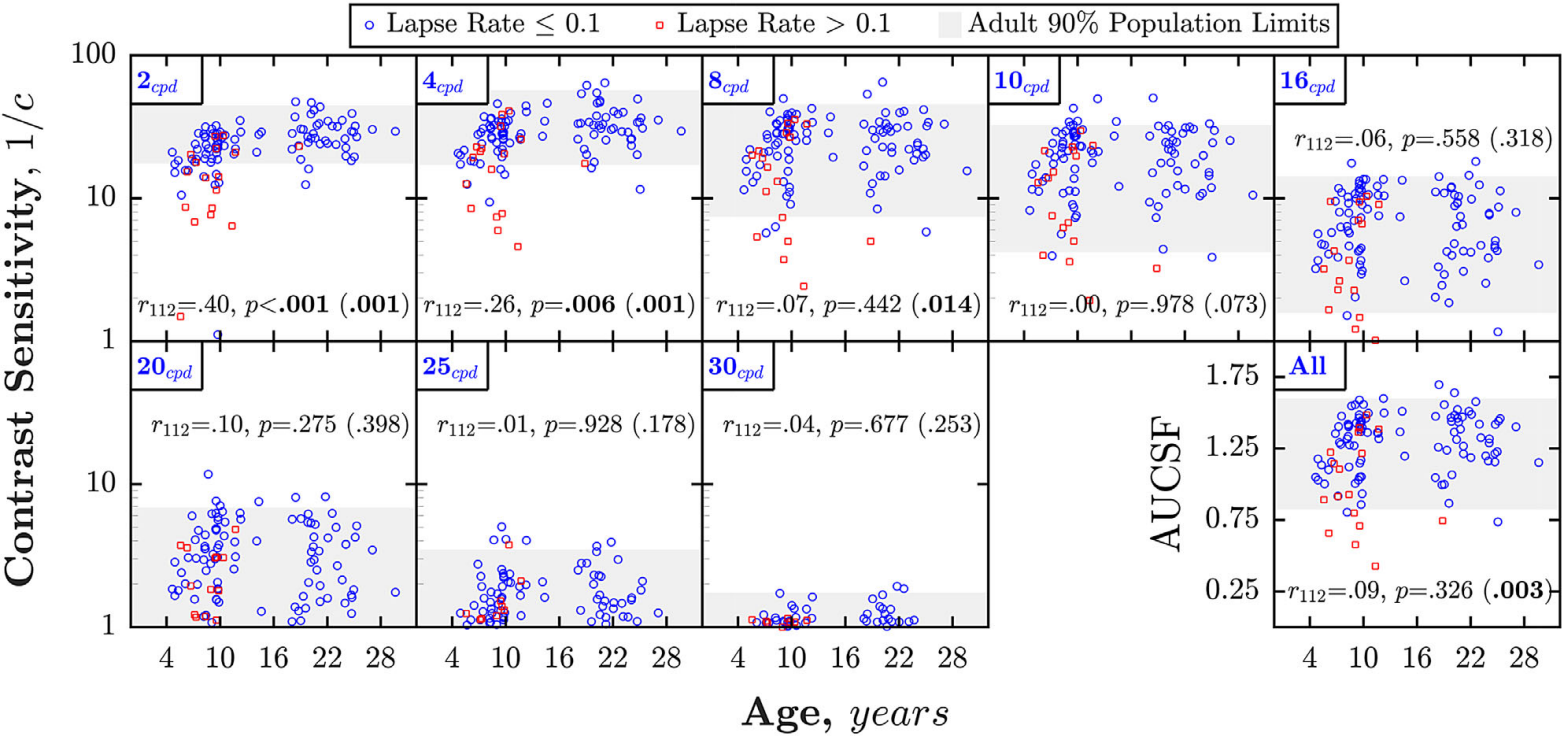
Data from experiment 1 of the present dataset, showing CS as a function of age, at each of the eight spatial frequencies tested (the original test frequencies in the case of the staircase method, and by inference from the fitted CSF with the ML method). Each marker represents a single observer (multiple values mean-averaged within observers). Red squares highlight those observers who exhibited lapse rates > 10%. Statistics indicate the result of Spearman Rho partial correlation between age and CS, with mean lapse rate as a controlling variable. The value in parentheses indicates the *p* value if an ordinary Spearman Rho correlation was performed (no control for lapse rates; see Table 2 for *r* values). The final panel shows equivalent data for a single overall summary measure (area under the curve, computed for each individual subject, and given in log_10_ units).

As detailed above, both previous and current data agree that, up to 4 cpd, CS increases by approximately 0.3 log_10_ between 4 and 18 years of age (a doubling of CS). To put the size of this developmental effect in context, Figure 2 shows CS for individual observers in experiment 1 of the new data set. From this, it can be seen that the developmental effect was small relative to interindividual variability. Thus, even at the lowest frequency (2 cpd), where the developmental effect appeared greatest, the mean difference in CS between adults and children aged 4.0 to 7.0 years (log_10_ CS = 1.45–1.11 = 0.34) was less than the range of CS values among normally sighted adults (95th–5th percentile: 1.65–1.24 = 0.41; gray shaded region of Figure 2), with most children performing as well as at least some adults. Likewise, across all ages, the amount of individual variance explained by age alone (*r*^2^) was only 29%, falling to just 16% when a more rigorous analysis was performed that controlled for lapse rates (see below).

**Figure 3.**
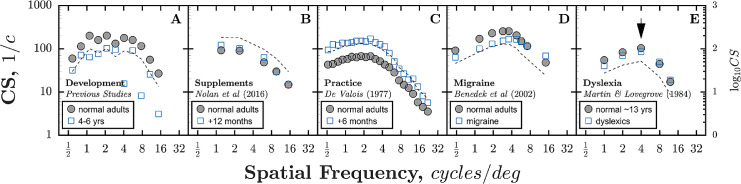
Comparison of developmental data (mean of all previously published studies listed in Table 1) with analogous data from non-developmental studies (De Valois, 1977; Martin & Lovegrove, 1984; Benedek et al., 2002; Nolan et al., 2016). Black dashed lines indicate the predicted change from normal adults given a 0.3 log_10_ unit increase/decrease in CS. Note that for ease of comparison, CS values for Martin & Lovegrove (1984) were uniformly scaled downwards (by dividing the reported logCS values by an arbitrary value of 2), as the values reported were atypically high.

Outside of the developmental literature, a 0.3 log_10_ CS change is similar to the magnitude of change observed in adult participants following practice (Figure 3C) or in patients experiencing migraine (Figure 3D). Similarly, Bradley and Freeman (1982) reported a 0.3 log_10_ CS change could be produced by asking two adults to variously concentrate or respond casually.

### Are any changes uniform across the CSF, or frequency specific?

On aggregate, prior studies appeared to indicate a relatively uniform increase in CS (see Figure 1C,D). However, several individual studies (see Figure 1A) reported selective changes at low frequencies only (Bradley & Freeman, 1982; Adams & Courage, 2002). Such frequency-specificity was observed in the present data also. Thus, as can be seen in Figure 1 (panels C, D, red squares), the greatest changes were observed at ≤ 4 cpd, whereas above 4 cpd the changes observed were small and statistically nonsignificant (see Figure 2).

To formally assess the interaction between age and spatial frequency, the following linear mixed effects (LME) model was run (Wilkinson notation): “log_10_ CS attenuation ∼ frequency * age + (1|participantID).” The results were analyzed using an analysis of variance (Welch–Satterthwaite method). Note that this analysis is conceptually similar to running a repeated measures ANOVA on the data summarized by the red squares in Figure 1C,D, but an LME model has the benefit of being able to tolerate the fact that participants were presented with slightly different spatial frequencies in experiments 1 and 2. There was a significant effect of age (*F*_(1, 172)_ = 20.2, *p* ≪ 0.001; CS attenuation decreasing with age), a significant effect of spatial frequency (*F*_(1, 700)_ = 29.9, *p* ≪ 0.001; CS attenuation decreasing with spatial frequency), and — crucially — a significant interaction of age and spatial frequency (*F*_(1, 703)_ = 11.6, *p* < 0.001), with a smaller effect of age on CS attenuation at higher spatial frequencies (see Figure 1D). Put simply, any changes in CS with age occurred primarily at lower spatial frequencies (≤ 4 cpd), and as evidenced by the post hoc correlations in Table 2, were smaller or nonexistent above this point.

As shown in Figure 3, a number of nondevelopmental studies have also reported selective changes to the CSF at low frequencies only. Thus, dietary supplements of macular carotenoids have been reported to induce small increases in CS at spatial frequencies 1.2 to 6 cpd (Nolan et al., 2016) (see Figure 3B), whereas extended practice has been reported to produce even more marked improvements at all spatial frequencies, but particularly those ≤ 4 cpd (De Valois, 1977) (see Figure 3C). Conversely, small deficits in CS at spatial frequencies 0.48 to 3.58 cpd have been observed in adult migraine patients (Benedek et al., 2002) (see Figure 3D), and CS deficits have been reported between 1 and 4 cpd in 12-year-old dyslexic boys (Martin & Lovegrove, 1984) (see Figure 3E; although subsequent investigations have tended to indicate a more uniform deficit, possibly indicative of general inattentiveness; Stuart, McAnally, & Castles, 2001). The mechanisms underlying these changes remain unknown, and so cannot help us understand the developmental effect reported in the present study (see Figure 3A). However, such observations provide convergent evidence that sensitivity losses in the low-frequency range of the CSF can be dissociated.

### Can some or all of the changes be explained by “non-visual” factors, such as boredom or inattentiveness?

**Figure 4.**
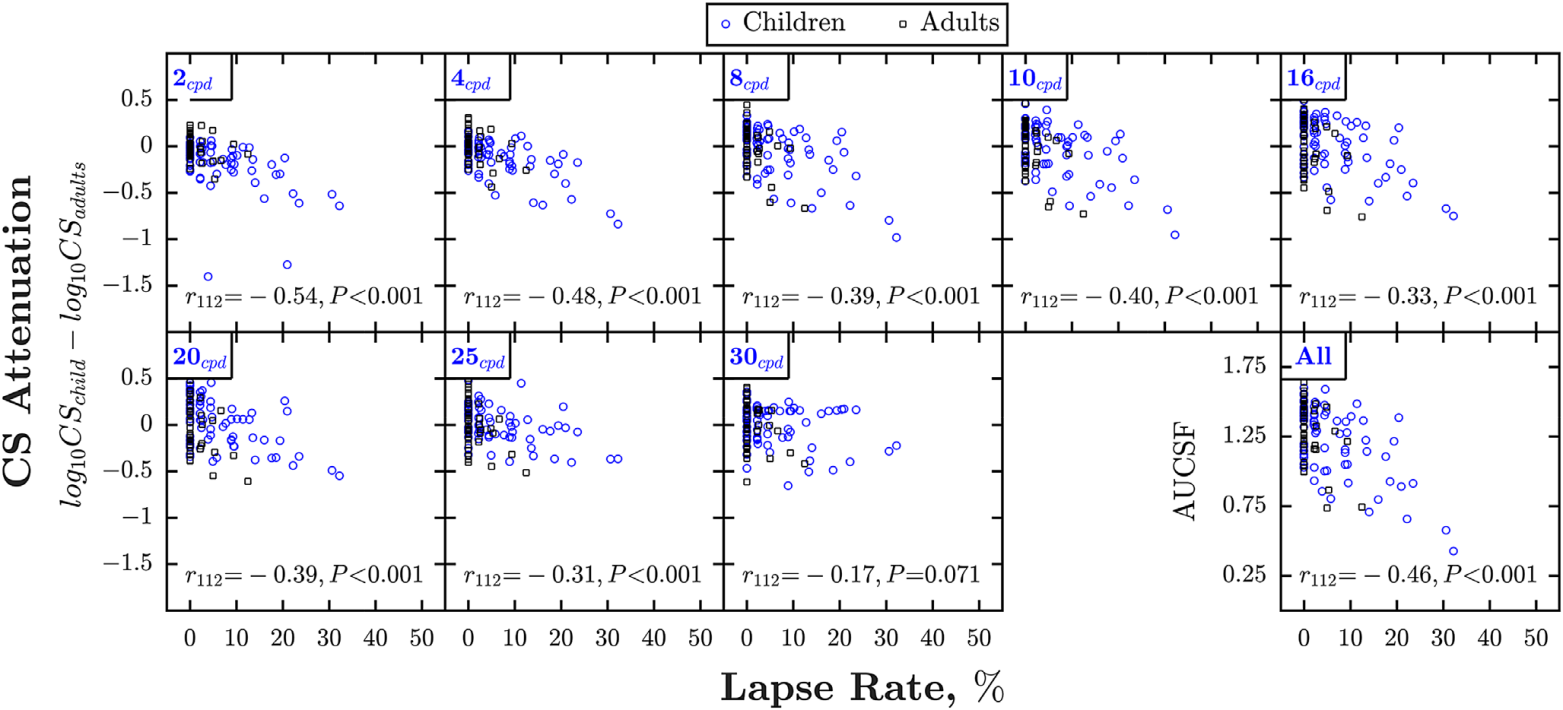
Contrast sensitivity as a function of lapse rate in experiment 1. Same format as Figure 2. Statistics indicate the result of ordinary Spearman's Rho correlations.

Higher lapse rates are known to be associated with higher (poorer) psychophysical thresholds in children (Westall et al., 1992; Wightman & Allen, 1992; Witton, Talcott, & Henning, 2017; Jones, 2018; Manning, Jones, Dekker, & Pellicano, 2018). This was the case with the present data also. Lapse rates were higher in children (mean {CI_95_} = 6.3 {4.7–8.3} %) than adults (mean {CI_95_} = 1.4 {0.7–2.4} %), and, as shown in Figure 4, lapse rates were negatively correlated with CS at every spatial frequency except 30 cpd (where performance was near floor for all observers). To what extent can this association explain the developmental changes observed in Figure 2? First, we note by inspection of Figure 2 that many of the children (and adults) that fell outside the adult 90% population limits (gray shaded regions) were those with lapse rates greater than 10% (red squares). Second, to more formally estimate the impact of lapses, the correlations between age and CS (shown in Figure 2 and Table 2) were re-run with and without controlling for lapse rates. When controlling for lapse rates, the amount of individual variability explained by age (*r*^2^) decreased from 29.2% to 16.0% at 2 cpd, from 16.8% to 6.8% at 4 cpd, and from 5.3% to 0.5% at 8 cpd (and ceased to be statistically significant). These correspond to relative reductions in explained variance of between 45% and 91%. Likewise, when using the Area Under the Curve of the CSF (AUCSF: an overall summary measure of CS) as the dependent measure, the amount of individual variability explained by age decreased from 7.3% to 0.8%, and again ceased to be statistically significant. A similar pattern emerged if we simply excluded those individuals with mean lapse rates greater than 10% (*N* = 17), with explained variance decreasing at all frequencies (and for AUCSF), and the correlation at 8 cpd becoming nonsignificant (see Table 2 for statistics).

Taken together, this suggests that at least half of the developmental effect is due to “non-sensory” (procedural) factors. The residual age-related changes in CS observed at low spatial frequencies may be genuine variations in visual function, or due to additional non-visual factors not captured by the present data (see Discussion).

## Discussion

### Main findings

From 4 to 18 years of age, the CSF improves (at an exponentially decaying rate) by approximately 0.3 log_10_ units (a doubling of CS), with 90% of the change from 4 years complete by 12 years. The size of the age effect is in many respects small: being less than the individual variability between normally sighted adults, and similar in magnitude to the effects of practice (De Valois, 1977), concentration (Bradley & Freeman, 1982), or migraine (Benedek et al., 2002) reported elsewhere (and far smaller than the > 1 log_10_ [i.e. tenfold] change observed during infancy; Norcia, Tyler, & Hamer, 1990; Peterzell, Werner, & Kaplan, 1995). Further, in the present data, age alone explained less than one sixth of individual variability (16%), with most children performing as well as some adults (i.e. falling within adults’ 90% population limits). Contrary to some previous studies (see below), our present data strongly indicated that development is frequency-specific, with changes occurring primarily around or below the peak of the CSF (≤ 4 cpd). A significant interaction between age and spatial frequency was observed, and above 4 cpd (and after controlling for lapses), no significant changes in CS were observed across any of the ages tested (4.7 to 29.7 years), suggesting that sensitivity to mid and high frequencies is fully mature by early childhood. At least half — and potentially all — of the changes observed were explained by non-visual (i.e. procedural/cognitive) factors. Across spatial frequencies, the amount of individual variability explained by age decreased by between 45% and 91% (relative reduction) when controlling for individual differences in lapse rates, and an apparent change in overall sensitivity (AUCSF) ceased to be statistically significant when controlling for lapse rates (see Table 2).

### Comparison to previous literature

At lower spatial frequencies (≤ 4 cpd), there was good agreement between the present data and previous studies, both in terms of the size and shape of the developmental trajectory.

Above 4 cpd, we observed no significant changes in CS. This is contrary to the overall pattern of previous data (see Figure 1), but is in keeping with a number of individual studies that reported no change (Bradley & Freeman, 1982; Adams & Courage, 2002), or less change (Gwiazda, Bauer, Thorn, & Held, 1997; Beazley, Illingworth, Jahn, & Greer, 1980; Benedek, Benedek, Kéri, & Janáky, 2003) at high spatial frequencies. It is also consistent with the wider developmental literature, which indicates that resolution acuity (the rightmost limit of the CSF) is fully developed by approximately 6 years (Catford & Oliver, 1973; Mayer & Dobson, 1982; Ellemberg, Lewis, Hong Liu, & Maurer, 1999; Leat, Yadav, & Irving, 2009), as well as with a range of nondevelopmental studies that have reported selective changes to the CSF at low frequencies only (see Figure 3).

Why some previous studies observed more uniform CSF changes is unknown. However, a uniform CSF shift can be indicative of general inattentiveness/noncompliance (e.g. in a small subset of children). In which case, we would predict that where group-average CSFs have been reported, the developmental effects would be attenuated (in general, and at high frequencies in particular) if more robust summary statistics were used (e.g. group-medians rather than group-means, which are liable to be skewed by small numbers of statistical outliers (Huber, 2011; Jones, 2019)).

Relatedly, our data highlighted that it is important to control for non-visual factors (e.g. lapses in concentration in a subset of children), which can otherwise exaggerate differences in CS between children and adults. This is consistent with previous observations that CSFs (Bradley & Freeman, 1982; Abramov et al., 1984) (and other psychophysical measurements (Westall et al., 1992; Wightman & Allen, 1992; Witton, Talcott, & Henning, 2017; Jones, 2018; Manning, Jones, Dekker, & Pellicano, 2018)) can be confounded by lapses in concentration, and is in keeping with the relatively long and demanding nature of a rigorous CSF assessment (and in spite of our concerted efforts to keep participants motivated in the present study, see Methods). This highlights the need to account for age-related variations in concentration (e.g. when setting clinical norms for visually healthy performance), and suggests that it may be worthwhile to explore the use of more engaging, “gamified” ways of delivering CS assessments to young children (Abramov et al., 1984; Wang et al., 2017; Elfadaly et al., 2020).

### Potential mechanisms

As detailed above, a large proportion of the observed change in CS could be attributed to age-related differences in task engagement. We can only speculate what causal mechanisms may underly the residual age-related changes in CS observed at low frequencies (≤ 4 cpd) after controlling for lapse rates; however, a number of plausible explanations exist.

**Non-visual (procedural) factors*.***One possibility is that the developmental effect is an artifact of further non-visual factors not already captured in our present data (i.e. random measurement error in the quantification of lapse rates, or transient lapses in concentration not detectable by our gross lapse rate metric (Jones, 2018; Jones et al., 2020)). For example, it might be that some children younger than approximately 12 years were more liable to become tired or distracted, or required more practice than adults to fully master the task. This “non-visual hypothesis” is the explanation largely favored by Abramov et al. (1984) and Bradley & Freeman (1982). Thus, Bradley and Freeman (1982) reported anecdotally that the 0.3 log_10_ CS change observed in children could be recapitulated in adults by asking them to concentrate to respond quickly and casually. Similarly, Abramov et al. (1984) concluded that CSFs at 6 to 8 years were “probably at adult levels,” despite observing empirically higher thresholds, after noting that “when we observe children performing these tasks, it is quite clear that, though highly motivated, they are not as attentive to near-threshold stimuli as are adults.” Against this stands the fact that differences between children and adults were observed only at low spatial frequencies (i.e. whereas one would intuitively expect a loss of motivation to result in a uniform downward shift of the whole CSF (Stuart, McAnally, & Castles, 2001)). Furthermore, the developmental effect was well conserved across different psychophysical methods (experiment 1; see Supplementary Results), and remained when only earlier or later trials were considered (see Table 2), and when participants received additional practice (experiment 2). Furthermore, as discussed in the Methods section, great lengths were taken to ensure that children understood the task and remained motivated and engaged throughout testing.

None of these arguments are conclusive, however. For example, it is conceivable that differences in some property of the stimulus (e.g. fewer numbers of cycles) or in how low-frequency data is processed in the brain (see below), mean that the task demands greater sustained attention at low frequencies. If so, there may be some interaction between stimulus properties and task-demands that results in performance being disproportionately affected by a loss of concentration at low frequencies. Alternatively, it might be that the slope of the psychometric function varies with spatial frequency, and the psychometric algorithms used were better suited for measuring higher spatial frequencies (i.e. a shallower slope would require a greater number of sub-asymptotic responses to estimate threshold, given a fixed adaptive step size, and so may be less robust to lapses in concentration).

In short, it would be premature to rule out non-visual factors as an explanation of all CSF differences between children and adults.

**Retinal physiology*.***During the first year of life, CS increases at both high and low spatial frequencies (Atkinson, Braddick, & Braddick, 1974; Harris, Atkinson, & Braddick, 1976; Atkinson, Braddick, & Moar, 1977; Banks & Salapatek, 1981; Atkinson & French, 1983; Movshon & Kiorpes, 1988; Riddell et al., 1997), and these changes are due in part to anatomic changes at the level of the retina (Banks & Crowell, 1993). It has been suggested previously that changes in the photoreceptor mosaic may likewise explain why the CSF continues to develop in later childhood (Ellemberg, Lewis, Hong Liu, & Maurer, 1999). This explanation is inconsistent, however, with the selective changes in low-frequency CS observed in the present study. It is also inconsistent with previous data from histology and optical coherence tomography (OCT), which suggest that the retina is largely mature by 5 years (Vajzovic et al., 2012) (although subtle differences have been observed until 13–16 years).

**Lateral inhibition*.***A third possibility is that the difference between pre-adolescent children and adults represents a change in lateral inhibition (LI). Thus, the dip in CS at low frequencies is widely thought to reflect lateral inhibitory processes in mammals and invertebrates (although see Banks & Salapatek, 1981), and the development of LI has been suggested as an explanation of why this low-frequency dip emerges in human infants after 3 months of age (Atkinson, Braddick, & Braddick, 1974; Atkinson, Braddick, & Moar, 1977). Children's poorer CS at low spatial frequencies might therefore reflect greater lateral inhibition in children. Convergent evidence for this hypothesis could be sought by examining the development of other quintessentially LI-related phenomena, such as Mach Bands, Hermann grid illusions, or the Ebbinghaus Illusion (although the exact role of lateral inhibition in these particular phenomena is debatable (Bach, 2009; Kingdom, 2014), and any effects, if they are LI-based, may depend on LI at different levels of the visual system). However, where such published data exist, they are not consistent with increased lateral inhibition in early childhood. If anything, the opposite has been indicated (Kaldy & Kovacs, 2003; Hudak, Iakab, & Kovacs, 2013).

**Magnocellular pathway*.***A fourth possibility is that the developmental effect reflects changes in the magnocellular (M) pathway (Benedek, Benedek, Kéri, & Janáky, 2003). Thus, it is often suggested that the anatomically larger cells in the M-pathway are predominantly responsible for the encoding of low-frequency spatial information (Merigan & Eskin, 1986; Lennie, 1993). This is supported by the fact that when the M-pathway is selectively saturated by a pulsed luminance signal, a marked deterioration of CS is observed psychophysically at spatial frequencies ≤ 4 cpd only (Leonova, Pokorny, & Smith, 2003). (Conversely, when the parvocellular (P) pathway is selectively saturated by a steady luminance signal, no such reduction is observed.)

By this logic, even greater developmental deficits would be observed for a quintessential M-pathway (“high temporal frequency, low spatial frequency, low luminance”) task, such as the detection of a flickering, low-spatial frequency grating. Flicker sensitivities have been reported for unpatterned fields of light, and have been found to actually develop many years earlier than static CSFs, both in human-children (Ellemberg, Lewis, Hong Liu, & Maurer, 1999; Braddick & Atkinson, 2009) and macaques (Stavros & Kiorpes, 2008) –contrary to the predictions of the M-pathway hypothesis. However, spatiotemporal CSFs are thought to develop at a different rate to full-field flicker sensitivity (Braddick & Atkinson, 2009), and when Benedek, Benedek, Kéri, & Janáky (2003) presented scotopic grating stimuli, they observed a pronounced low-frequency developmental effect in children tested, which the authors interpreted as consistent with M-cell hypothesis. Overall, evidence for or against an M-pathway hypothesis remains sparse, and somewhat inconsistent.

Further, against this M-pathway hypothesis more generally, it should be noted that although individual magnocellular neurons are more sensitive than P neurons at low frequencies, the two systems as a whole exhibit highly overlapping CSFs (Merigan & Maunsell, 1993), and data following selective-lesioning indicate that the P-pathway may even be more sensitive to low-frequency static stimuli (Skottun, 2000). Likewise, there also appears to be a large overlap in the range of luminances over which neurons in P and M pathways respond, and the suggestion that the M pathway dominates vision under scotopic conditions remains controversial (Merigan & Maunsell, 1993).

### Conclusions

1. From 4 to 18 years of age, the CSF improves (at an exponentially decaying rate) by approximately 0.3 log_10_ units (a doubling of CS), with 90% of the change from 4+ years complete by aged 12 years.
2. The size of the effect was smaller than individual variability between normally sighted adults, and similar in magnitude to the effects of practice, concentration, or migraine reported elsewhere. Further, age alone explained less than one sixth of individual variability (16%), and most children performed as well as some adults (fell within the 90% population limits for adults).
3. Previous developmental data are conflicted, but on aggregate indicate a roughly uniform increase in CS with spatial frequency. In the present data, however, development appeared to be frequency-specific, with changes occurring primarily around or below the CSF peak (≤ 4 cpd). Above 4 cpd (and after controlling for lapses), no significant change in CS was observed across any of the ages tested (4.7 to 29.7 years), suggesting that sensitivity to mid and high frequencies is fully mature by early childhood.
4. At least half — and potentially all — of the changes observed could be explained by non-visual factors (e.g. lapses in concentration). When controlling for differences in lapse rates, the amount of individual variability explained by age at individual spatial frequencies decreased by between 45% and 91% (relative reduction), and an apparent change in overall sensitivity (AUCSF) ceased to be statistically significant.
5. Possible neural mechanisms for the change in CS at low frequencies are discussed, including changes in lateral inhibition or magnocellular pathways. However, we cannot rule out residual non-visual (“procedural”) factors (e.g. transient lapses in concentration not detectable by our gross lapse rate metric), and we believe this remains the most parsimonious explanation.

## Supplementary Material

Supplement 1

Supplement 2

## Footnotes

1 The weighted median of a sample is the 50% weighted percentile. It can be computed by sorting the set of numbers by value, finding the middle weight (the weight whose running sum crosses 50% of the total weight), and then finding the value associated with that weight. It is a robust estimator of central tendency, that can, for example, account for measurements of varying precision within a sample.
