# Supplementary material for: Development of the spatial contrast sensitivity function (CSF) during childhood: Analysis of previous findings and new psychophysical data
Source: J Vis. 2020 Dec 4;20(13):4. doi: 10.1167/jov.20.13.4 (PMC7718811; doi:10.1167/jov.20.13.4)
Supplement: Supplement 1 [file jovi-20-13-4_s001.pdf]

Supplemental Material

Development of the spatial contrast sensitivity function (CSF) during childhood: Analysis of previous findings and new psychophysical data

Mahtab Farahbakhsh, Tessa M. Dekker, Janette Atkinson, Oliver J. Braddick, & Pete R. Jones

1. Supplemental *Methods*

1.1. Participants

The distribution of participant ages in Experiment 1 is shown in **Supplemental Figure S1**.

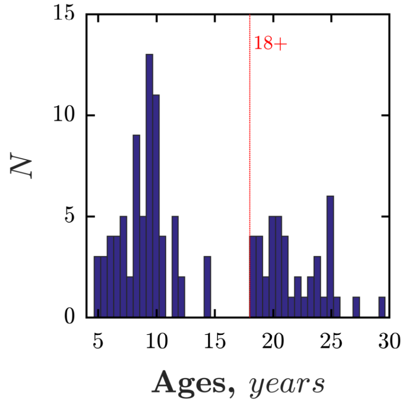

Fig S1. Histogram of participant ages in Experiment 1.

1.2. Staircase algorithm

The Staircase procedure consisted of 8 independent adaptive tracks, each of which estimated Contrast Sensitivity (CS) at a particular spatial frequency. Spatial frequencies were fixed at: $\langle 2, 4, 8, 10, 16, 20, 25, 30 \rangle$ cpd in Experiment 1, and: $\langle 0.5, 1, 2, 3, 4, 8, 15, 30 \rangle$ cpd in Experiment 2. Within each adaptive track, Michelson contrast was varied using a down-1 up-2 Weighted Staircase, which targets the 66.7% correct point on the psychometric function. Step sizes were multiplicative, and decreased every four trials, going: $\langle 3, 2, 1.5, 1.25 \rangle$, and remaining at 1.25 thereafter (e.g., a step size of 2 meant that the contrast halved/doubled after a correct/incorrect response). Each adaptive track started two steps away from the expected threshold at that frequency, as determined by piloting. The number of trials was not fixed, and each adaptive track continued until 19 reversals had occurred (Mean N_{trials}=47, including additional catch trials that were not used when determining reversals). Contrast threshold, c, was calculated by geometric-mean-averaging the last 8 reversals. CS was defined as the reciprocal of this value.

The final output was a vector of eight CS values – one per spatial frequency. During analysis, the following CSF was numerically fitted to these values:

$$CS = \begin{cases} 1/exp_{10}\left(log_{10}(G_{max}) - log_{10}(2)\left(\frac{log_{10}(f) - log_{10}(F_{max})}{log_{10}(2\beta)/2}\right)^2\right) & \text{if } f > F_{max} \\ 1/exp_{10}\left(log_{10}(G_{max}) - log_{10}(2)\left(\frac{log_{10}(f) - log_{10}(F_{max})}{log_{10}(100)/2}\right)^2\right) & \text{otherwise} \end{cases}, \quad (1)$$

where G_{max} is peak gain (contrast sensitivity), F_{max} is peak spatial frequency (in cpd), and β is the rate of CS fall-off at high spatial frequencies (full width half maximum, in octaves). The action of these three parameters is illustrated previously in Farahbakhsh et al (2019)[1]. Note that this formulation of the CSF represents a modified version of the log-parabola model recommended previously by Lesmes[5] and others[9]. For simplicity, however, and since low-frequency attenuation tends to be reduced for extrafoveal stimuli anyway[4], the rate of fall-off at low frequencies was fixed at a relatively shallow value of 100; a value which appeared to give a good account of our present data (see **Supplemental Figure S2**), and which allowed us to reduce the free parameters in our model to 3. Note that there is no general consensus on the 'correct' CSF model, and several competing CSF models have been suggested[9] (often with 4+ parameters). Determining the optimum function was outside the scope of the present work, however, and collecting sufficient data in individual children would be a non-trivial task.

The fitting of **Eq 1** was performed using a bounded nonlinear minimization procedure (MATLAB's fminsearchbnd routine), with parameters constrained thus: [$2 \leq G_{max} \leq 100$], [$2 \leq F_{max} \leq 30$], and [$0.5 \leq \beta \leq 9$].

Using the Staircase procedure, the median {IQR} duration for each CSF assessment was 12.0 {10.9, 13.3} mins in children, and 10.8 {9.9, 12.0} mins in adults.

1.3. ML algorithm

In Experiment 1 (only), 78 participants also performed a QUEST+[8] maximum likelihood (ML) procedure similar to the 'quick CSF' (qCSF)[2,5,7] in which contrast ($0 - 1$) and spatial frequency ($2 - 30$ cpd) are adapted simultaneously, in order to directly fit a single overall CSF. The underlying model that QUEST+ attempted to fit consisted of a Weibull psychometric function relating the expected proportion of a correct response, $P_{correct}$, to stimulus contrast, c, thus:

$$P_{correct} = \gamma + (1 - \gamma - \lambda)\left[1 - exp_e(-10^{\varphi(log_{10}c - log_{10}\alpha)})\right]. \tag{2}$$

The lower asymptote, γ, upper asymptote, λ, and slope φ, were fixed parameters, with values 0.25, 0.1, and 3 respectively. The lower asymptote ('guess rate') value of 0.25 was known a priori (i.e., in an mAFC paradigm $\gamma = 1/m$). The upper asymptote and slope values were set based on pilot data, and were only intended as approximations. The values were similar to those used elsewhere in the literature (e.g., $\varphi = 2$, $\lambda = 0.04$ in Lesmes and colleague's qCSF method[5]), but were somewhat greater to reflect the poorer concentration and/or lower sensitivity of some children. Note that, given the nature of QUEST+, λ and φ could have also been made free parameters, but this would have been impractical, given the additional data/trials required to constrain a five-dimensional parameter space (though see [[6]]). The key 'threshold' parameter, α, was a free parameter that varied with spatial frequency in accordance with the following 3 parameter CSF:

$$\alpha = \begin{cases} 1/exp_{10}\left(log_{10}(G_{max}) - log_{10}(2)\left(\frac{log_{10}(f) - log_{10}(F_{max})}{log_{10}(2\beta)/2}\right)^2\right) & \text{if } f > F_{max} \\ log_{10}(G_{max}) & \text{otherwise} \end{cases}. \tag{3}$$

Note that unlike **Eq 1** there was no fall-off of sensitivity at low frequencies included in this model. This is unlikely to have made a substantive impact, since the ML algorithm was only used in Experiment 1, where no stimuli below 2 cpd were presented. However, in order to facilitate easier comparison, the data were refitted post hoc using **Eq 1**. At this point, each parameter was also refitted with more steps ($N = 80$ per parameter), in order to minimize quantization error. For further details regarding how the ML model was implemented (incl. stimulus domain, parameter domain, response domain) see [[1]]. For MATLAB code, see [[3]]. No explicit priors were included in the model.

Using the ML procedure, the median {IQR} duration for each CSF assessment was 7.6 {6.2, 9.9} mins in children, and 6.3 {5.7, 6.9} mins in adults.

2. Supplemental *Results*

2.1. Experiment 1

Supplemental Figure S2 shows CSF data for 35 (of 114) individuals for both psychophysical methods, sampled uniformly across the tested age range. To assess overall changes in CS, we also computed Area Under the CSF (AUCSF) as a summary measure of performance. In general there was good agreement between the two psychophysical methods [*Spearman's Rho*, comparing AUCSF in those 47 observers who performed both methods; $r_{45} = 0.92$, $p < 0.001$], and across repeated runs of the same method [both $p < 0.001$; see **Supplemental Figure S4** for full analysis]. Accordingly, for all following analyses, CSFs were combined within observers (by mean-averaging the fitted CSF parameters), to give one single 'best-estimate', per observer.

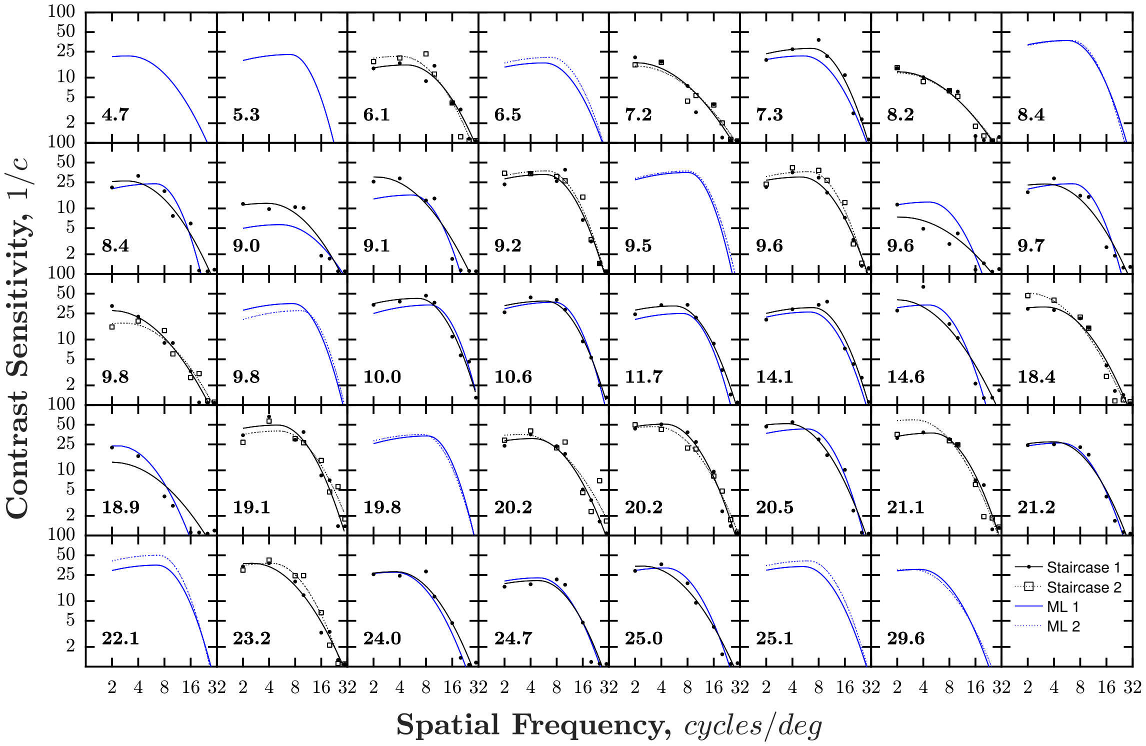

Fig S2. Individual CSFs (Experiment 1). Data are shown for 35 (of 114) individuals, sampled from youngest (4.7 years) to oldest (29.6 years). Participants were sampled uniformly by age, from 0th, 2.9^{th}, 5.9^{th}, ..., 100th percentile. Lines show fits to **Eq 1** for the Staircase (black lines) and ML (blue lines) procedure. For the Staircase method, markers show the raw threshold estimates for each block. For the ML method, fits were computed based on individual trials, which could vary in both contrast and spatial frequency.

As shown in **Supplemental Figure S4**, there was a possible trend towards AUCSF increasing with age [*Spearman's Rho*; Staircase: $r_{81} = 0.16$, $p = 0.153$; ML: $r_{76} = 0.22$, $p = 0.052$]. However, this developmental effect appeared to be an artifact of non-visual (procedural) factors (i.e., low estimated sensitivity in a small handful of younger children with elevated lapse rates; **Supplemental Figure S4**, red markers). As such, the association between AUCSF and age was no longer significant if individuals with high mean lapse rates ($> 10\%$) were excluded [Staircase: $r_{68} = 0.07$, $p = 0.569$; ML: $r_{67} = 0.12$, $p = 0.308$], or if a partial correlation was performed, with mean lapse rate as a controlling variable [Staircase: $r_{81} = 0.01$, $p = 0.917$; ML: $r_{76} = 0.07$, $p = 0.533$]. (Though note that some older children and adults also exhibited high lapse rates, indicating that catch trials may be an important feature when testing children of any age.) In short, we found no compelling evidence for a change in overall CS during childhood, as well as confirmatory evidence that CSF estimates are liable to be affected by motivational factors such as boredom or failures of sustained attention (i.e., as reflected by lapse rates).

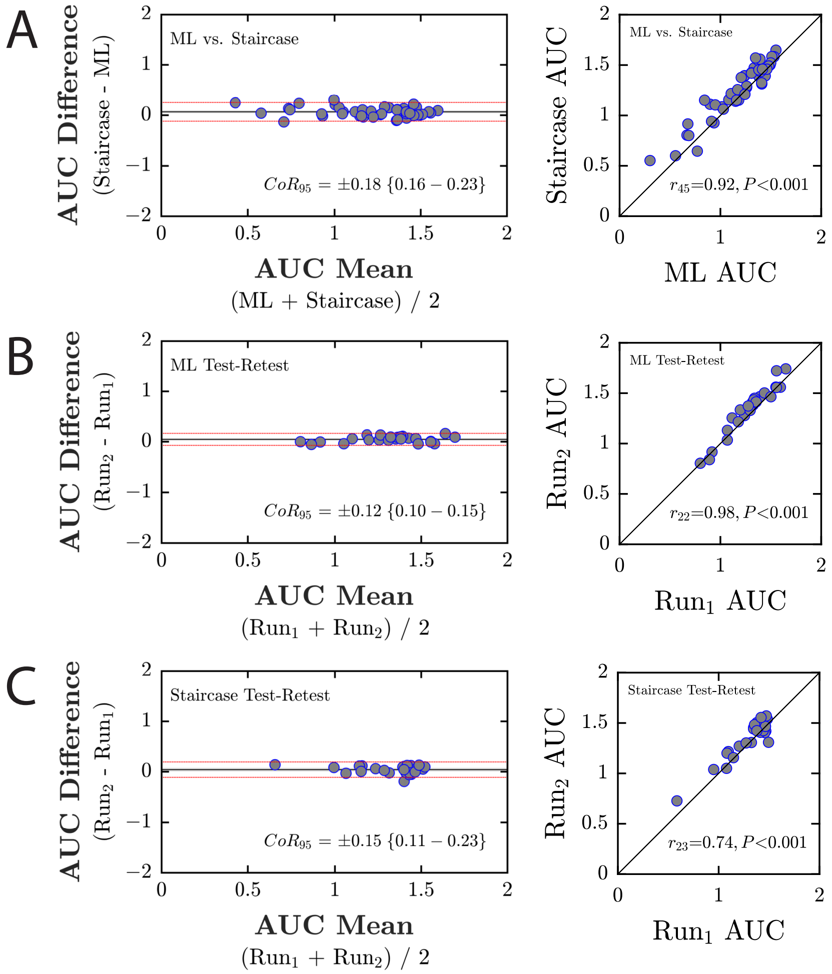

Fig S3. Comparison of the two psychophysical methods employed in Experiment 1. There was good agreement between the ML data and Staircase data (***A***). Overall, the level of agreement between the two methods was similar to the level of agreement within repeated applications of a single method (***B, C***).

Inspection of group-median CSFs for different age-groups (**Supplemental Figure S5**), however, indicated a possible, selective increase in sensitivities at low spatial frequencies only. Thus, the youngest children ($4 - 7$ years) appeared to exhibit lower sensitivities than adults at frequencies ≥ 4 cpd, and, by inspection, sensitivities did not appear to match adult-like levels until around $10 - 13$ years.

2.2. Experiment 2

Experiment 1 indicated a small, selective developmental change in CS at low spatial frequencies (≤ 4 cpd). To confirm and extend this finding, in Experiment 2 we made further measurements, in 34 new observers, using additional low-frequency stimuli. Observers in this experiment also underwent more extensive practice to try to minimize non-visual (procedural) confounds.

Supplemental Figures S6, **S7** show CSF data for individuals, and group-medians, respectively. In general, sensitivities were higher than in Experiment 1. This is likely due to the larger and/or more central stimulus in Experiment 2, and possibly the more extensive practice. The relative pattern of development was consistent, however, with Experiment 1. The 8-9 and 10-13-year-old groups exhibited a lower CS than adults for stimuli ≤ 4 cpd, but not for higher frequencies (note the 95% confidence intervals in **Supplemental Figure S6**). The performance of 13-15-year-olds

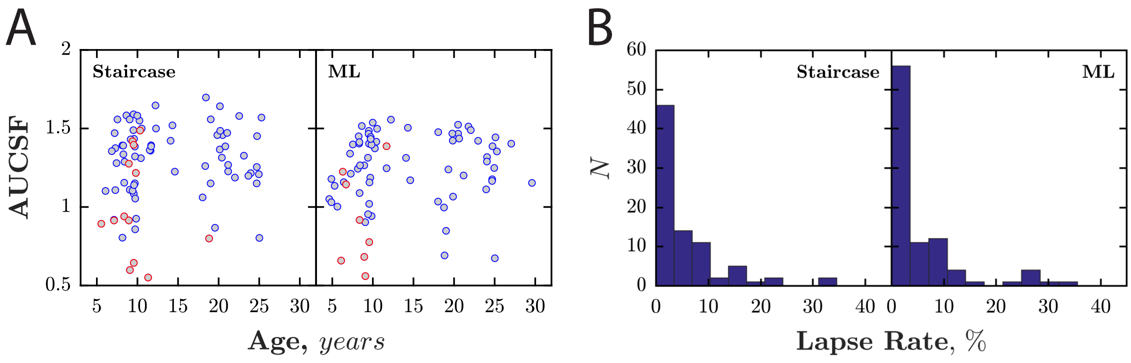

Fig S4. (***A***) Area Under the CSF (AUCSF) as a function of age. Each marker represents a single individual (AUCSF values mean-averaged within each observer, in instances where the observer completed two assessments using the same method). Red markers indicate observers with lapse rates greater than 10%. (***B***) Distribution of lapse rates in Experiment 1. Values greater than 10% correspond to red markers in panel A.

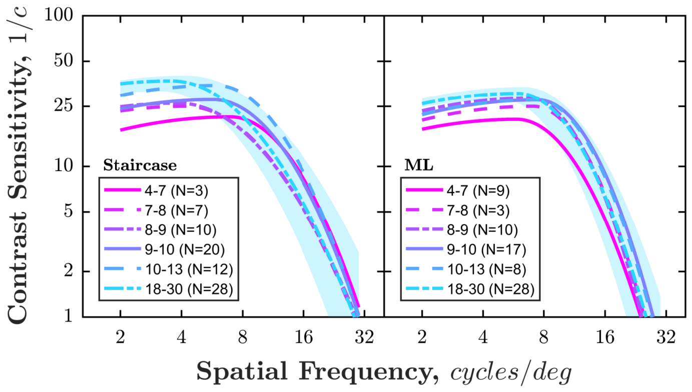

Fig S5. Group-median CSFs (Experiment 1). Lines show group-median CSFs for each age group. These were computed by fitting each individual's data using **Eq 1**, and then median-averaging the three fitted parameters (G_{max}, F_{max}, β). The blue shaded region indicates bootstrapped 95% CIs for adults (not shown for other age groups, to avoid clutter). Note that the age groups shown here are unequal in terms of N years, in order to maintain a reasonable number of participants per group.

was indistinguishable from adults, again suggesting that the developmental trajectory is complete by around 13 years. (Though it should be noted that, while there is insufficient data for any statistical analysis, the four 9-10-year-olds in Experiment 2 appeared to perform roughly as well as adults, again possibly highlighting the small size of the developmental effect relative to the amount of individual variability).

In short, Experiment 2 appeared to confirm that at low spatial frequencies (≤ 4 cpd) CS is not adult-like until adolescence (~13 years), though the size of this developmental effect appears small.

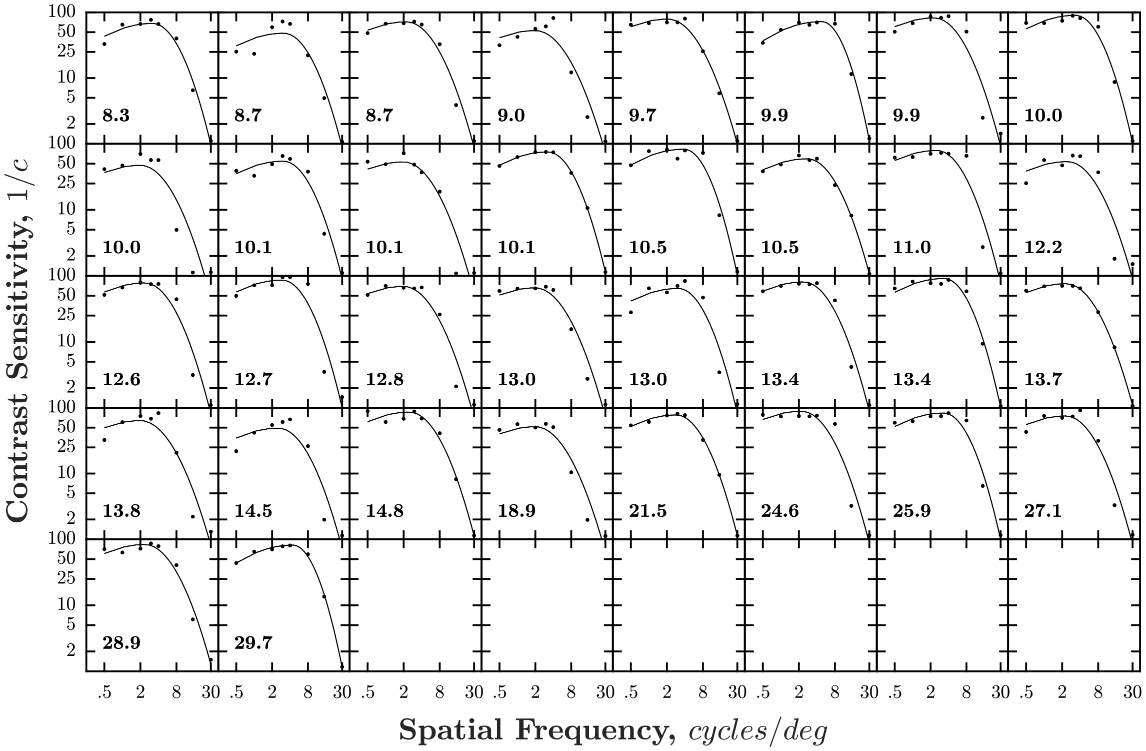

Fig S6. Individual CSFs for Experiment 2, shown in the same format as the data for Experiment 1 (**Supplemental Figure S2**).

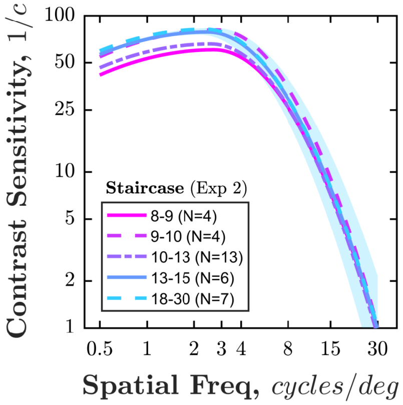

Fig S7. Group-median CSFs for Experiment 2, shown in the same format as the data for Experiment 1 (**Supplemental Figure S5**).

Supplemental References

[1] M. Farahbakhsh, T. M. Dekker, and P. R. Jones. Psychophysics with children: Evaluating the use of maximum likelihood estimators in children aged 4 – 15 years (quest+). *Journal of Vision*, in press.

[2] F. Hou, C.-B. Huang, L. Lesmes, L.-X. Feng, L. Tao, Y.-F. Zhou, and Z.-L. Lu. qcsf in clinical application: Efficient characterization and classification of contrast sensitivity functions in amblyopia. *Investigative ophthalmology & visual science*, 51(10): 5365–5377, 2010.

[3] P. R. Jones. Questplus: A matlab implementation of the quest+ adaptive psychometric method. *Journal of Open Research Software*, 6(1), 2018.

[4] L. M. Katz, D. M. Levi, and H. E. Bedell. Central and peripheral contrast sensitivity in amblyopia with varying field size. *Documenta Ophthalmologica*, 58(4):351–373, 1984.

[5] L. A. Lesmes, Z.-L. Lu, J. Baek, and T. D. Albright. Bayesian adaptive estimation of the contrast sensitivity function: The quick csf method. *Journal of Vision*, 10(3):17–17, 2010.

[6] N. Prins. The psi-marginal adaptive method: How to give nuisance parameters the attention they deserve (no more, no less). *Journal of Vision*, 13(7):3–3, 2013.

[7] R. Rosén, L. Lundström, A. P. Venkataraman, S. Winter, and P. Unsbo. Quick contrast sensitivity measurements in the periphery. *Journal of vision*, 14(8):3–3, 2014.

[8] A. B. Watson. Quest+: A general multidimensional bayesian adaptive psychometric methodwatson. *Journal of Vision*, 17(3): 10–10, 2017.

[9] A. B. Watson and A. J. Ahumada. A standard model for foveal detection of spatial contrast. *Journal of vision*, 5(9):717–740, 2005.
